# Using mobile phones to improve community health workers performance in low-and-middle-income countries

**DOI:** 10.1186/s12889-020-8173-3

**Published:** 2020-01-13

**Authors:** Anam Feroz, Rawshan Jabeen, Sarah Saleem

**Affiliations:** Department of Community Health Sciences, The Aga Khan University, Stadium Road, PO Box 3500, Karachi, Pakistan

**Keywords:** Community health workers, Mobile phones, Performance, Productivity, Low-middle-income countries

## Abstract

**Background:**

In low-and-middle-income countries community health workers are the core component of the PHC system as they act as a liaison between the communities and the healthcare facilities. Evidence suggests that the services offered by these workers have helped in the decline of maternal and child morbidity and mortality rates and the burden of communicable and non-communicable diseases. However, the coverage and the overall progress towards achieving the SDG targets is very sluggish. The recent consensus concerning this current pace of progress, is that it relates to financial and human resources constraints. CHWs are overburdened as they are expected to accomplish more although they may not obtain the required support to perform their duties. The health systems of LMICs, have given very little attention to the work environment of CHWs; which has negatively affected CHWs productivity, and quality of services. This debate is intended to explore the potential of mobile phone technology in LMICs for improving CHWs performance and effectiveness.

**Discussion:**

To improve CHWs productivity, some studies involved the use of mobile phones for data collection and reporting, while other studies used mobile technology for patient to provider communication, patient education, CHWs supervision, and monitoring and evaluation. A wide range of benefits exists for using mobile phones including reduction in CHWs workload, improvement in data collection, reporting and monitoring, provision of quality healthcare services, supportive supervision, better organization of CHWs tasks and improvement in community health outcomes. However, a number of studies suggests that CHWs encounter unique challenges when adopting and using mobile health solutions for health service delivery such as, lack of CHWs training on new mHealth solutions, weak technical support, issues of internet connectivity and other administrative challenges. Future research efforts should be directed to explore health system readiness for adopting sustainable mHealth solutions to improve CHWs workflows in LMICs.

**Conclusion:**

Future research efforts and policy dialogue should be directed to explore health system readiness for adopting sustainable mHealth solutions to improve CHWs workflows in LMICs.

## Background

The notion of primary healthcare (PHC) is fundamental to improve population health outcomes and ensure effective health service delivery [1]. PHC has underwent tremendous evolution, starting from Alma Ata Declaration to the latest Astana Declaration, a renewed commitment to PHC, to achieve universal health coverage (UHC) and sustainable development goals (SDGs) [2]. Despite prominent progress in the last four decades, there are still gaps in PHC delivery in many low-income and middle-income countries (LMICs) due to diverse contextual and health system related challenges [3]. In LMICs, community health workers (CHWs) are the core component of the PHC system as they act as a liaison between the communities and the healthcare facilities. These workers provide a range of promotive, preventive and curative, and rehabilitative healthcare services in the underserved areas. The health advice provided by CHWs is highly valued and accepted by the population as these workers are often the first and the only point of contact for community, especially for pregnant women and newborn [4].

In LMICs, CHWs have been greatly involved in various PHC activities initiated by public health department, non-governmental organizations (NGOs) and donor agencies [5]. These initiatives include maternal, newborn and child health program (MNCH), nutrition program, vaccination program, communicable disease control program, health emergency response activities, disease surveillance, etc. Thus, there is a wide scope of services offered by the CHWs to the community, ranging from provision of safe delivery, and counseling on breast-feeding, management of uncomplicated childhood illnesses, to preventive health education on communicable and non-communicable diseases (NCDs). Evidence suggests that the services offered by these workers have helped in the decline of maternal and child morbidity and mortality rates and have also assisted in decreasing the burden of communicable and non-communicable diseases [6]. However, the coverage and the overall progress towards achieving the SDG targets is very sluggish [7].

The recent consensus concerning this current pace of progress, especially in the LMICs, is that it relates to financial and human resources constraints. CHWs are overburdened as they are expected to accomplish more although they may not obtain the required support to perform their duties well, such as supportive supervision, training, remuneration, transportation, supplies and equipment, and respect from the community and health system [8]. There is no approved scope of practice for CHWs and the ongoing question of how many duties an individual worker should effectively perform is still unanswered. Logically, when the workload is pushed beyond a certain extent, the performance of the worker gets compromised [8]. Alongside, political corruption and the problem of increasing “ghost workers” in health labor markets of LMICs overstrain the health systems and imposes a significant burden on the community health workforce [9]. The health systems of LMICs, have given very little attention to the work environment of CHWs; which has negatively affected CHWs productivity, quality of services, job satisfaction levels, and, eventually the effectiveness of community-based programs. This calls for an urgent solution that has an ability to create an enabling work environment for attaining high levels of CHWs performance in LMICs, thereby improving the quality of PHC services.

What might stimulate progress? An apparent and accessible solution is coupling efforts of CHWs with mobile phone technology to ensure that CHWs have a manageable workload, in terms of number of duties and households to cover, an organized workflow for carrying out assigned tasks, a reasonable geographical distance to reach, and the needed support from a trained supervisor. Worldwide, population is getting access to the mobile phones, as they are inexpensive, user friendly, and do not need much literacy and proficiency. According to the digital 2019 report, there are 5.11 billion unique mobile users worldwide, with a penetration of 67%. With regard to internet usage, there are 4.39 billion internet users in 2019 globally with penetration of 57% [10]. In LMICs, cell phone penetration has surpassed over 90% in the recent years and the mobile internet connectivity is around 40% [11, 12]. The numbers are expected to increase and surpass the world population in the years ahead. This new innovation and near ubiquity of mobile phone uptake around the world and especially in LMICs offer novel opportunities for integrating technology into CHWs workflow to improve CHWs work environment, and productivity. This debate is intended to explore the potential of mobile phone technology in resource-constrained LMICs for improving CHWs performance and effectiveness, thus improving the quality of PHC services.

## Main text

### Feasibility of using mobile phones for improving CHWs work environment

Various LMICs have advocated the feasibility of using mobile technology to ensure advancements in PHC by improving the work environment and workflow of CHWs. To improve CHWs productivity, some studies involved use of mobile phones for data collection and reporting, while other studies used mobile technology for patient to provider communication, patient education, clinical decision making, CHWs supervision, and monitoring and evaluation. Few countries have also integrated mobile health to address logistical issues that hamper CHWs performance such as covering long geographical distances, time constraints, and unavailability of transport and supplies. Most studies conducted in LMICs involved the use of short message service (SMS) and voice communication to improve CHWs performance [13, 14]. While, some studies used unique mobile phone functions to ensure CHW interaction with communities and physicians [15, 16]. These mobile phone functions include, multimedia messaging service (MMS), Interactive voice response (IVR), video clips, images, audio clips, mobile phone camera, and digital forms. Generally, the use of basic mobile phone was preferred in LMICs to improve CHWs workflows [13, 17, 18].

In rural Ghana, CHWs were trained to collect postpartum hemorrhage outcome data using basic mobile phones. CHWs reported 3.1% cases of PPH during the 90-day period, which indicated improvement in CHWs performance [13]. In rural Uganda, community-based peer health workers (PHW) used phones to call and text higher level providers to communicate patient-specific clinical information. The study found out that the mHealth support intervention used by PHWs resulted in improvements in patient care and logistics [14]. In Indonesia, the midwife mobile phone project was implemented in 15 health centers, involving 223 midwives (MWs). The study reported that the technology proved to be appropriate for MWs, as it addressed issues of data entry, time-sensitivity, health knowledge and training and MW mobility to remote areas [16]. In Tanzania, the study evaluated the impact of short message service (SMS) reminders to improve CHW promptness of CHW visits. The study found out that the escalating reminder system is very beneficial in which the SMS reminder is sent directly to the CHW before notifying the CHW’s supervisor. The intervention resulted in an 86% reduction in the number of days a CHW’s routine visits were overdue (9.7 to 1.4 days) [18]. In Columbia, a prospective, randomized controlled study was conducted where clinical guidelines to diagnose and treat pediatric and adult medical conditions were presented on mobile phones in a structured interactive workflow using rich media (text, audio/voice, and images/video). This mobile rich media job aids were used by CHWs in intervention group, whereas controlled group CHWs used traditional paper-based job aids. The study results indicated that the use of rich media job aids on small-format mobile platforms reduced errors related to diagnosis and management by an average of 33.15% (*p* = 0.001), thereby improving CHWs performance [15]. In rural India, CHWs receive resistance from pregnant women to change health seeking behaviors because of their limited education, training and socioeconomic status. These factors appear to affect rural maternal health system and decrease the motivation of CHWs. To address this issue, CHWs used short videos on mobile phones to persuade village pregnant women to utilize health services. The study results showed that the videos facilitated in engaging pregnant women in dialogue, improved rural maternal health system, and enhanced motivation and learning for CHWs [19]. In Bangladesh, Manoshi Project has piloted the use of smartphones to improve capacity of health workers and thereby enhancing slum dwellers’ access to maternal, neonatal and child health. The project has equipped CHWs with mobile phones to collect vital patient data and also to assess risk on the basis of an embedded algorithm. This new innovation assists CHWs in record keeping, reporting, monitoring, and providing basic maternal and neonatal health services [20]. In Nepal, a pilot intervention undertaken in Gulmi District, whereby all health workers in the peripheral health facilities were provided with a free phone number to call General Practitioner in the District Hospital. The intervention aimed to increase appropriate referral, and increase connectivity between the District center and peripheral health facilities. Overall, the health workers and patients were positive about the intervention and mentioned that they felt more supported, and the consultation helped them to make decisions in emergency cases [21].

In LMICs, the mobile technology has been used by CHWs to address a range of health issues related to maternal and child health, sexual and reproductive health, family planning, HIV/AIDS, general health, acute respiratory infections, infectious diseases and injury and trauma [22]. This is evident from the literature that the use of mHealth technology has helped CHWs in data collection and reporting, communication with high level providers, and in organization of their workflow, which has eventually improved CHWs productivity in various primary healthcare programs.

### Benefits of using mobile phones for CHWs

Given the ubiquity of mobile phones in LMICs, the new innovation of mobile health hold promise and great benefits for supporting CHWs by improving their performance, workflow and work environment. A wide range of benefits exists for using mobile phones for community health workers. These include reduction in CHWs workload, improvement in data collection, reporting and monitoring, provision of quality healthcare services, supportive supervision, better organization of CHWs tasks and improvement in community health outcomes. In Kenya, a quasi-experimental post-test design was conducted to improve CHWs performance using a mHealth tool. The study findings indicate that CHWs who used mHealth tool (*n* = 196) captured and transmitted higher percentages of monthly cases without missing information; compared to CHWs (*n* = 199) who used paper-based data collection tool. Moreover, CHWs who used mHealth tool were found to be more satisfied in terms of their performance [23]. In rural Tanzania, mobile health application was developed to: 1) guide CHWs as they counsel family planning clients 2) collect feedback of clients on quality of care 3) train CHWs on how to use mobile application 4) implement a pay-for-performance mechanism for CHWs who register and follow up with clients and 5) use data to convince governments on areas that need actions. After finishing the intervention, the study found that the mHealth solution got three major achievements: 1) improvements in CHWs workflow and perceived quality of family planning services 2) Increase in client registration and follow-up and 3) 95% of clients used family planning during the project period [24]. CommCare software has been developed by researchers at Washington and California University to support CHWs in a variety of tasks including, illness screening (TB, Malaria), making follow-up visits, providing adequate information on safe drinking water and family planning, tracking and registering new births and deaths, etc. This application is an example of point-of-care support, which allowed CHWs to organize their workflow, track clients follow-up appointments, retrieve healthcare standards and protocols, and collect and timely report health data [25]. Researchers believe that coupling mobile phones with CHWs will help spread health education messages to people and thus improve health seeking behaviors of communities. CHWs could use mobile phones (reminder text messages, calls) to influence community about health lifestyle changes such as physical exercise, avoidance of risky sexual behaviors, adherence to TB treatment, routine visits to doctor to receive antenatal and postnatal care, etc. [22]. In Saraya, Senegal the qualitative study was conducted to explore health workers experience of using wireless technology for community case management (CCM) of malaria. The study reported that health workers greatly valued the use of mobile phones as it helped them address issues of training, stock management, reporting, and transportation [26]. In Uganda and Mozambique, a study was conducted to explore views of CHWs on the potential role of mHealth in their work delivering integrated community case management (iCCM) of children. The research found that mobile health improved overall CHWs performance, motivation, efficiency, communication with supervisor and other CHWs, and addressed issues of frequent travelling [3]. Some mHealth interventions supported CHWs in data collection and reporting; while other mHealth interventions tend to improve their workflow and performance, and quality of the services provided by CHWs.

### Challenges of using mobile phones for community health workers

Use of mHealth solutions for health service delivery can potentially improve CHW performance and strengthen quality of services. However, a number of studies suggests that CHWs encounter unique challenges when adopting and using mobile health solutions for health service delivery. These include lack of CHWs training on new mHealth solutions, weak technical support, issues of internet connectivity and other administrative related challenges. Ngabo et al. found that that regular training of FHWs reduced the error rate for data entry from 54% at the start of the program to 8% over the course of 1 year [27]. Thus, it is fundamental to provide sufficient initial and ongoing training to support the transition of workflow from a paper-based system to a digitized system [28]. Weak technical support in new mHealth projects greatly influence the functioning of the intervention and outcome of the study. In two districts of Zambia, Community Health Management Information System (C-HMIS) was developed using simple mobile phones for use by 40 CHWs and their 20 supervisors. CHWs used the mobile phones to submit data on cases seen, managed, and referred. Whereas, supervisors tracked CHWs’ reported cases, and provided feedback on their referrals. The study found that C-HMIS was feasible for the provision of real-time community-based health information to all levels of the health care system in Zambia; however ongoing technical support is needed to address the hardware and software related challenges CHWs face in their day-to-day work process [29]. A formative research conducted in Uganda and Mozambique reported that CHWs encounter some challenges such as the difficulty in sending SMS in local language, poor network coverage, concern regarding equipment maintenance and problems with phone charging [17]. Additionally, factors such as age, education level, years of experience and technology proficiency levels also affected the CHW readiness to adopt new mHealth practices. A systematic review conducted by Agarwal et al., reported that front line health workers (FHWs) with low level of literacy often find it difficult to adapt new mHealth interventions. Therefore, user-friendly mHealth solutions should be integrated in routine workflow of CHWs to avoid withdrawal using freeform text challenging [28]. Besides, some main barriers to adoption of mobile health solutions include poor sustainability of pilot projects, high investment, operational, and maintenance costs and other management related issues. Unfortunately, many mHealth initiatives are often unsustainable pilot projects that not only fail to ‘scale up’ meaningfully, but also are concluded once initial funding is exhausted. High investment, operational and maintenance costs associated with setting up mHealth services in community setting, also pose a major challenge for health systems of LMICs [30]. While integrating mobile health solutions in CHWs workflow, another significant challenge faced by CHWs are management related challenges. A mobile phones system was implemented in rural Nepal to collect health surveillance data; however, it did not reach its fullest potential due to several management related challenges such as, leadership transitions, poor process design and a lack of consistent vision of how to operationalize the data [31]. The researchers concluded that for any mHealth intervention to be successful in a low-resourced settings, institutional buy-in, appropriate and actionable data collection techniques, and effective process management are essential and should be taken in to consideration [31].

## Conclusion

There is an ample evidence base suggesting that the use of mHealth interventions is essential to improve CHWs performance and work environment in LMICs. The mobile technology has been used by CHWs to address a range of health issues related to maternal and child health, sexual and reproductive health, family planning, HIV/AIDS, general health, acute respiratory infections, infectious diseases and injury and trauma. Mobile phones have proven effective for CHWs as it has improved routine CHWs workflows such as, data collection and reporting, patient to provider communication, patient education, decision making, supportive supervision, CHWs monitoring and evaluation. There are some challenges concerning the implementation and sustainability of mobile phone interventions for CHWs in LMICs, including lack of CHWs training on new mHealth solutions, weak technical support, issues of internet connectivity, administrative and management related issues, poor sustainability of pilot projects, and high investment, operational, and maintenance costs of equipment. Given the gaps in the literature, future research efforts and policy dialogue should be directed to explore health system readiness for adopting sustainable mHealth solutions to improve CHWs workflows in LMICs. Further, there is a need to conduct a qualitative research with stakeholders to understand how mobile health solutions can be better designed and implemented in LMICs to ensure improved CHWs performance in communities.

## Data Availability

Not Applicable.

## Abbreviations

CCM: Community case management
C-HMIS: Community Health Management Information System
CHWs: Community health workers
FHWs: Front line health workers
ICCM: Integrated community case management
LMICs: Low-income and middle-income countries
MNCH: Maternal, newborn and child health program
MWs: Midwives
NCDs: Non-communicable diseases
NGOs: Non-governmental organizations
PHC: Primary healthcare
PHW: Peer health workers
SDGs: Sustainable development goals
SMS: Short message service
UHC: Universal health coverage
